# Fluorometric Imaging for Early Diagnosis and Prognosis of Rheumatoid Arthritis

**DOI:** 10.1002/advs.201902267

**Published:** 2019-12-01

**Authors:** Jeong Heon Lee, Sang Youn Jung, G. Kate Park, Kai Bao, Hoon Hyun, Georges El Fakhri, Hak Soo Choi

**Affiliations:** ^1^ Gordon Center for Medical Imaging Department of Radiology Massachusetts General Hospital and Harvard Medical School Boston MA 02114 USA; ^2^ Division of Rheumatology Department of Internal Medicine CHA Bundang Medical Center CHA University Seongnam 13496 South Korea; ^3^ Department of Biomedical Sciences Chonnam National University Medical School Gwangju 501‐746 South Korea

**Keywords:** near‐infrared fluorescence, progressive imaging, rheumatoid arthritis, targeted fluorophores

## Abstract

Early diagnosis and monitoring of disease progress are of significant importance in the effective treatment of rheumatoid arthritis (RA), because the continuing inflammation can lead to irreversible joint damage and systemic complications. However, applying imaging modalities for the prognosis of RA remains challenging, because no tissue‐specific guidelines are available to monitor the progressive course of RA. In this study, fluorometric imaging of RA is reported using bioengineered targeted agents of the blood vessel, bone, and cartilage in combination with the customized optical fluorescence imaging system. Separate but simultaneous tissue‐specific images of synovitis, cartilage destruction, and bone resorption are obtained from a mouse model of RA, which allows quantification of the prognosis of diseases at each stage. Thus, the fluorometric imaging of RA by using tissue‐specific contrast agents plays a key role in the systemic treatment of RA by monitoring structural damage and disease progression.

## Graphical abstract

Near‐infrared fluorescence imaging is a fast, inexpensive, nonionizing, yet accurate and quantitative method for the early diagnosis and prognosis of rheumatoid arthritis. Combined with targeted contrast agents, separate but simultaneous tissue‐specific imaging of synovitis, cartilage destruction, and bone resorption can be obtained. This strategy allows quantitative biomedical imaging of articular tissue in response to the progressive course of rheumatoid arthritis.

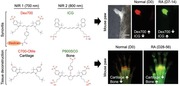

## 1 Introduction

Rheumatoid arthritis (RA) is a serious, progressive autoimmune disease that results in chronic inflammation with permanent disability of multiple joints.^1^ RA has an insidious onset and principally affects synovial tissues with symmetrical pain and swelling of peripheral joints.^1^, ^2^, ^3^ Of note, this inflammatory arthropathy is different from degenerative arthritis (i.e., osteoarthritis), because the clinical symptoms and disease progression are different.^4^ The clinical diagnosis of RA is generally established by physical examination, joint radiographs, serological tests, and, more recently, by musculoskeletal ultrasonography and magnetic resonance imaging (MRI).^5^, ^6^ Although the anatomical imaging modalities have been improved substantially over the past 10 years, patients and physicians still have to deal with limited information provided by unclear imaging during the progressive course of RA.^5^, ^6^ Furthermore, since the joint destruction is typically irreversible, molecular imaging in the clinical practice should focus on the synovial joint tissue in the early stages of RA.

Optical fluorescence imaging is a fast, inexpensive, and nonionizing modality that provides new opportunities for the effective diagnostic and prognostic evaluation of RA by assessing the altered level of molecular distortion.^7^, ^8^ Importantly, articular tissues, such as the synovial membrane, cartilage, and bone, have specific pathophysiological patterns throughout the course of RA. The prominent aspects of acute inflammation are hypervascularized synovitis, pannus invasion, marginal erosions, and osteopenia, whereas the chronic stage is associated with joint deformity, malalignment, and ankylosis.^1^, ^9^ In any stage, selective visualization of articular tissue can be a distinct diagnostic indicator in response to the progressive course of RA. However, the lack of tissue‐specific optical contrast agents has been the main limitation for early diagnosis and prognostic imaging of RA.

Recently, we developed novel near‐infrared (NIR; 650–900 nm) fluorophores that exhibit specific uptake in the cartilage, bone, and other vital tissues by virtue of structure‐inherent targeting.^10^, ^11^, ^12^ In addition, noninvasive optical fluorescence imaging with vascular perfusion agents, such as indocyanine green (ICG), has been used to monitor synovial inflammation in arthritic joints.^13^, ^14^ We therefore hypothesized that the selective fluorometric detection of articular tissue could provide diagnostic intervention in RA progression. The goal of this study was to assess RA tissue at each inflammatory stage and to monitor the pathophysiological outcomes, respectively and simultaneously, by using targeted NIR fluorophores and a dual‐channel optical imaging system. By normalizing the NIR signal readouts in the context of permissive scores, the progressive trajectory of RA could be evaluated and quantified.

## 2 Results

### 2.1 Targeted NIR Fluorophores and Imaging System for RA Disease Progression

**Figure 1:**
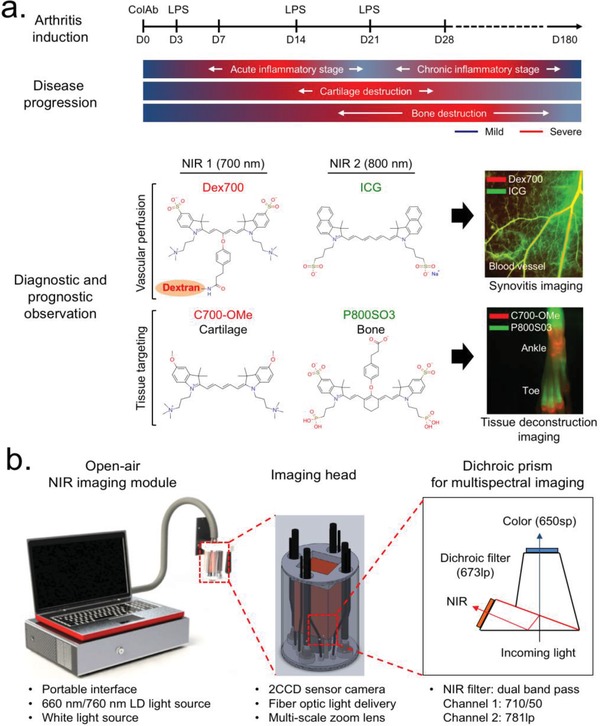
Overall strategy of imaging for the diagnosis and prognosis of RA. a) Preparation of a CAIA mouse model and dual‐channel NIR imaging along with RA disease progression (top), and chemical structures of the NIR fluorophores with the following outcome from an in vivo assessment (bottom). Red and lime green colors represent 700 and 800 nm NIR, respectively. b) Open‐air, intraoperative optical imaging system equipped three excitation light sources, a 2CCD camera, and multi‐scale zoom lens. Details of imaging head and dichroic prism‐based beam filtration for visible and NIR lights are described in the schematic drawing.

**Figure** **1**a illustrates the overall experimental design. The joint inflammation and tissue destruction in collagen antibody‐induced arthritis (CAIA) on DBA/1J mice was observed from the day of injection (day 0 (D0)) of anti‐type II collagen antibodies to over the period of chronic stage (D180).^15^ We divided the disease progression into three different indications in response to the physical appearance of subjects, such as synovial inflammation, cartilage destruction, and bone resorption. The inflammation can be further divided into three different stages (i.e., acute, chronic, and long‐standing stages), and such transitional stages were assessed by dual‐channel optical imaging with the following tissue‐specific NIR fluorophores (Figure 1a): 1) Dex700 (700 nm NIR)^16^ and ICG (800 nm NIR) were prepared to assess synovitis severity; and 2) C700‐OMe (700 nm NIR)^10^ and P800SO3 (800 nm NIR)^12^ were synthesized to monitor the destruction process of cartilage and bone, respectively. Both C700‐OMe and P800SO3 are composed of polymethine backbone and structure‐inherent targeting moieties.^10^, ^12^ All NIR fluorophores are high extinction coefficients and high quantum yields (QYs), which together minimize tissue autofluorescence and maximize the signal‐to‐background ratio (SBR; see Figure S1, Supporting Information). Physicochemical and optical properties of each NIR fluorophore are summarized in Table S1 (Supporting Information). Figure 1b shows the intraoperative optical imaging system equipped with the prism‐based 2CCD camera for multispectral NIR imaging. In vivo mouse imaging was fully assessable from the whole body (field of view = 6 cm) to each peripheral joint (field of view = 1 cm) at a 9 in. working distance (Figure S2, Supporting Information). The specific design of the three light sources (i.e., white light, 660 nm laser, and 760 nm laser) and filtration of emitted light made the aforementioned four NIR fluorophores amenable to targeted tissue imaging.^17^, ^18^

### 2.2 Imaging of Synovial Tissue

**Figure 2:**
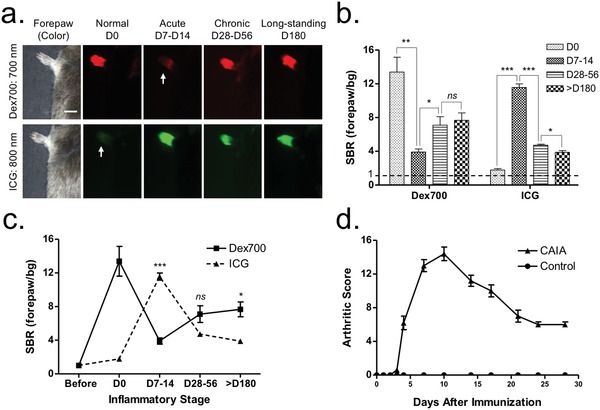
Noninvasive synovitis assessment in mice with CAIA. a) In vivo imaging of mouse forepaws after the intravenous injection of Dex700 (25 nmol, red color) and ICG (50 nmol, green color) 30 min prior to imaging. Scale bars = 1 cm. Images are representative of independent experiments (*n* = 3). Arrows indicate the clearance of each NIR fluorophore from the bloodstream. b,c) Quantitative scoring (SBR) charts of Dex700 and ICG for D180 in the inflammatory stages. SBR was calculated by the fluorescence intensity of the forepaw versus the background (bg) signal of the neighboring blank area (*n* = 3, mean ± s.d.; *P < 0.05, **P < 0.01, and ***P < 0.001). d) Clinical arthritis scores in the paws. The total score per mouse is 0–16.

We injected both Dex700 (700 nm NIR) and ICG (800 nm NIR) intravenously into the same mice with CAIA, which allowed independent, noninvasive, and real‐time assessments of vascular perfusion of the peripheral synovial tissues. We compared vascular permeability of each fluorophore in the mouse paws, corresponding to inflammatory stages (**Figure** **2**a–c; and Figure S3, Supporting Information). When intravenously injected, Dex700 (25 nmol; 0.5 mg kg^−1^) predominantly persisted in the bloodstream (*t*_1/2β_ = 80 h; SBR = 13) even 30 min post‐injection, whereas ICG (50 nmol; 1.2 mg kg^−1^) was mostly cleared out by the liver within 5 min. Interestingly, this pattern was reversed during the acute stage of inflammation (D7‐D14): Dex700 retention in the bloodstream was dramatically reduced at 30 min post‐injection, whereas ICG could highly visualize local hyperperfusion in the forepaw (SBR = 11). However, both perfusion agents remained in the vascular bed between the chronic stage and long‐standing stage with a moderate SBR of 4–7. This perfusion pattern of ICG corresponds to the clinical score of synovitis,^19^ which was maximized by soft tissue swelling in the acute stage (Figure 2d; and Figure S4, Supporting Information).

### 2.3 Imaging of Cartilage Destruction

**Figure 3:**
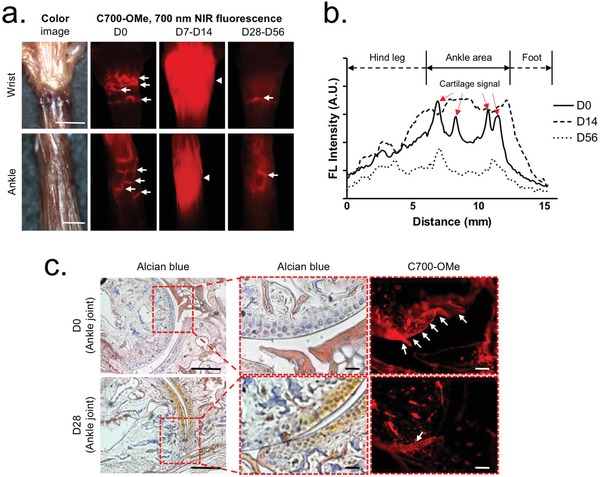
In vivo cartilage imaging using C700‐OMe over the progression of RA. a) 25 nmol (0.05 mg kg^−1^) of C700‐OMe was injected intravenously into mice with CAIA 2 h prior to imaging of the cartilage in the wrist and ankle. Scale bars = 3 mm. Images are representative of independent experiments (*n* = 3). b) A line profile chart of fluorescence intensity in each stage. c) Alcian blue and NIR imaging of resected ankle tissue from (a). Scale bars = 50 µm. Red pseudocolor was used for 700 nm NIR images. Arrows indicate the articular cartilage linings.

To determine the process of cartilage destruction, a 700 nm emitting cartilage‐targeted agent C700‐OMe was injected intravenously into mice with CAIA (25 nmol; 0.5 mg kg^−1^) 2 h prior to imaging.^10^ Articular cartilages in the wrist and ankle area were then exposed under the dual‐channel NIR imaging system at each inflammatory stage (**Figure** **3**a). On D0, the articular cartilage linings were clearly visualized, thereby showing multiple steep slopes in the line profile of fluorescence intensity (Figure 3b). Of note, as the hypertrophic synovial tissue expanded under the cartilage palisades (D7‐D14), C700‐OMe accumulated in the hypervascularized pannus nonspecifically, resulting in an undesirable high fluorescence covering the whole area. On D28‐D56, the CAIA mice presented with fully developed pathophysiologic features of arthritis with severe cartilage destruction, whereas C700‐OMe highlighted very limited cartilage linings with a faint slope profile and dwindled signal intensity.

We further performed histological evaluations of the destructed cartilage by using hematoxylin‐eosin and Alcian blue–Safranin‐O staining.^20^ After decalcification using ethylenediaminetetraacetic acid (EDTA), the ankle joint was cryosectioned and imaged using an epi‐fluorescence microscope. As shown in Figure 3c, the co‐staining of Alcian blue and Safranin‐O confirmed the presence of sulfated glycosaminoglycans (GAG) in the cartilage of D0, where strong NIR fluorescence was found. However, cartilage images of D28 represented markedly reduced signals of Alcian blue and C700‐OMe, where the cartilage matrix was largely degraded. The narrow joint space and partially fused tarsal bone were also observed as typical features of the chronic stage of RA (Figure S5, Supporting Information).^4^ The abnormal yellowish color from the image on D28 resulted from the predominant staining by Safranin‐O without Alcian blue. Taken together, the profile of C700‐OMe uptake represented the cartilage destruction process of mice with CAIA in real time, where the local concentration of cartilage matrix is reduced.

### 2.4 Imaging of Bone Destruction and Resorption

**Figure 4:**
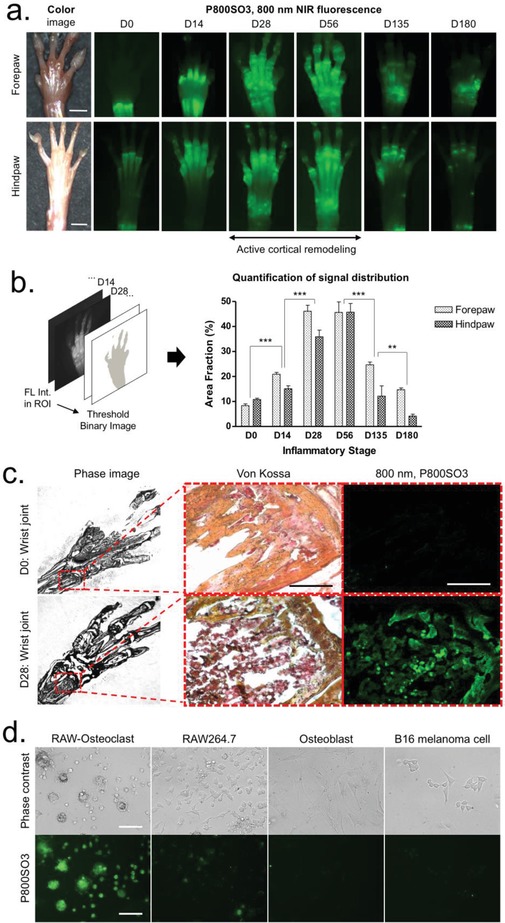
In vivo bone imaging using P800SO3 over the progression of RA. a) 25 nmol (0.05 mg kg^−1^) of P800SO3 was injected intravenously into mice with CAIA 24 h prior to imaging of bone in the forepaw and hindpaw. Scale bars = 3 mm. Images are representative of independent experiments (*n* = 3). b) The quantitative analysis of bone images by acquiring area fraction (%) of binary images and a signal distribution chart during the inflammatory stages. c) Von Kossa staining and NIR imaging of resected wrist tissue from (a). Scale bars = 100 µm. d) Static cell assay to identify specificity of P800SO3 among various cell types, including Raw264.7‐differentiated osteoclast, Raw264.7, osteoblast, and B16 melanoma cells. Scale bars = 50 µm. Lime green pseudocolor was used for 800 nm NIR images.

In parallel with cartilage imaging, we simultaneously assessed the process of bone destruction and resorption. Compared to other bone targeting agents, P800SO3 binds to bone minerals, such as hydroxyapatite (HA) and calcium phosphate (CP)^12^ (Figure S6, Supporting Information), and thus allows quantitative and ratiometric imaging of bone destruction based on changes in osteoblast and/or osteoclast activity. We injected 25 nmol (0.5 mg kg^−1^) of P800SO3 intravenously into the mice with CAIA 24 h prior to imaging, and the mice were sacrificed to expose bone tissue. At the normal stage of D0, P800SO3 showed higher accumulation in the epiphysis and radius compared to the diaphysis and phalanges (**Figure** **4**a). Notably, the specific uptake of P800SO3 extended over the metacarpal area on D14 in response to the progress of inflammation, followed by continuous distribution to the entire peripheral bones during the chronic inflammatory stage of D28‐D56. This phenomenon reflects the process of active cortical remodeling.^21^ In the late chronic stage of D135‐D180, specific fluorescence signals in the bone drastically decreased due to the low‐grade inflammation. This destruction process in peripheral bones was also confirmed by microcomputed tomography (CT) imaging, corresponding to the result of NIR fluorescence imaging (Figure S7, Supporting Information). Subsequently, we quantified the signal distribution in the region of interest (ROI) by calculating binary fluorescence signals (100% = total area), after setting the same threshold value in each experiment (Figure S8, Supporting Information). As presented in Figure 4b, the percentage (%) area fraction was correlated with the progress of RA. In particular, it reached the peak in the D28‐D56 stage (mean area fraction = 46%), where the local inflammation reached the maximum (chronic stage).

To determine the mechanism of action for this progressive uptake of P800SO3, we performed histopathologic examination using Von Kossa staining and static cell assay (Figure 4c,d).^22^ The region of extensive bone resorption was discolored dark red or brown in the wrist bone specimen, indicating dispersed calcium deposits and recruitment of immune cells (i.e., macrophages). This local biological change was reflected by the strong uptake of P800SO3 in the disease area (D14‐D56). Interestingly, several speckles were observed from the fluorescence image due to changes in the density of osteoclasts, which are actively involved in the process of bone resorption and mineral release. Surprisingly, as shown in Figure 4d, the cellular binding of P800SO3 reached the maximum in osteoclasts that were differentiated from cytokine‐activated macrophages. P800SO3 progressively accumulated in the entire peripheral bone area and area actively involved in the process of cortical remodeling, which together can be used for the prognosis of RA even in the early inflammatory stage.

### 2.5 Site‐Dependent Imaging of RA Progression

**Figure 5:**
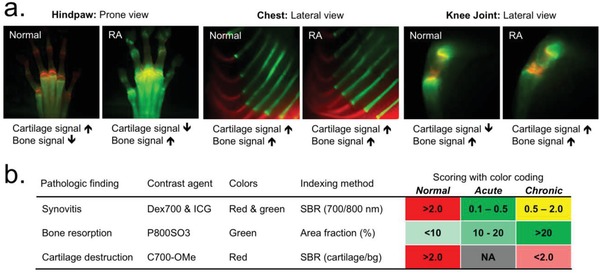
Real‐time dual‐channel intraoperative imaging of cartilage and bone. a) 25 nmol (0.05 mg kg^−1^) of C700‐OMe and P800SO3 was injected into the same mice with CAIA 2 h and 24 h prior to imaging, respectively. Cartilage and bone of central skeleton in the chest area, large joint in the knee area, and peripheral small joints in the hindpaw area were observed in the same RA model. Red and lime green pseudocolors were used for 700 and 800 nm NIR, respectively, in the color‐NIR merged images. b) Permissive scoring index for the prognosis of RA. The metric was based on the SBR and area fraction (%) of synovial inflammation, bone resorption, and cartilage destruction, and the color codes were provided for a convenient visual interpretation of inflammatory severity and disease progression.

We explored the progress of RA in the chest and knee joint areas by comparing them with the hindpaw peripheral joint, using dual‐channel imaging of C700‐OMe and P800SO3, simultaneously and in real time. P800SO3 (25 nmol; 0.5 mg kg^−1^) was injected into the mice with CAIA 24 h prior to imaging, followed by the injection of C700‐OMe (25 nmol; 0.5 mg kg^−1^) into the same animals 2 h prior to imaging. Under this condition, cartilage and bone tissues in the same animal could be unambiguously discriminated. As shown in **Figure** **5**a, remarkable changes in fluorescence intensity and distribution were observed only in the peripheral joint after co‐injection of cartilage and bone imaging agents. P800SO3 distributed into overall peripheral bones, including digits in the RA stage, while the strong uptake of C700‐OMe in normal cartilages gradually decreased during the progression of RA. Both cartilage and bone in the chest retained high signals in the RA stage (D28), and no specific tissue deformation was observed. In the knee joint, unlike peripheral tissues, the appearance of degenerative change seemed to be delayed, as evidenced by an enhanced cartilage signal and unvaried bone signal, and thereby the diagnostic significance of inflammation was uncertain. However, the epiphyseal bone of the knee joint was resorbed slowly without increasing on‐site osteoclastogenesis, which was observed using P800SO3 longitudinally during the course of RA over D180 (Figure S9, Supporting Information).

### 2.6 Permissive Scoring Index for RA Prognosis

Quantitative imaging‐based monitoring of biological activity and anatomical changes resulted in three independent data sets from a single RA carrier. We quantified the fluorescent information with numerical values to classify inflammatory and destructive changes in the mouse models of RA. As shown in Figure 5b, we developed a simple, activity‐based scoring index that can be used for RA diagnosis, particularly for identifying disease stages. The color code provides convenient visual interpretation of the disease severity with RA progression based on a quantitative metric of SBR and area fraction (%). For synovitis, the scale is 0.1–2.0 proportional to the SBR of 700 and 800 nm NIR fluorescence: 0.1–0.3, acute stage; 0.5–2.0, chronic stage; and >2.0, normal stage of inflammation. The bone signal was scored 0–20 by a fractional distribution of 800 nm fluorescence: <10, normal stage; 10–20, acute stage; and >20, chronic stage of RA. The cartilage signal was used to separate normal and chronic stages because of the nonspecific uptake in the acute RA stage: >2.0, normal stage; and <2.0, chronic stage of RA. Although the scoring system is yet rudimentary, the criteria underlying the combination of the three independent values can be useful for the diagnosis and prognosis of RA.

## 3 Discussion

Biomedical imaging has played an important role in the diagnosis of human diseases. However, early diagnosis and prognosis of disease are still a significant challenge due to the lack of targeted contrast agents. Using tissue‐specific contrast agents, we explored real‐time imaging of RA prognosis by selectively monitoring physiological changes in synovial membrane, cartilage, and bone with a multi‐channel imaging system. The strategy behind imaging of acute and chronic inflammation is tissue‐specific vascular permeability with RA progression. At the onset of RA, the inflammatory cells that migrate into the synovial membrane secrete various types of proinflammatory cytokines, chemokines, and proteolytic enzymes that affect the joint tissue and cells.^23^ This promotes extensive neoangiogenesis, leading to increased vascular permeability, fluid collection in the joint cavity, and subsequent hypervascularization.^24^, ^25^ The vascular leakiness in the acute inflammatory phase determined immediate Dex700 clearance from the blood vessels. In contrast, hypervascularized synovitis was assessed by ICG protein binding in the synovial space. Consequently, a complementary pattern was observed in two separate NIR channels in the normal and acute phases. Since vascular normalization takes time, concurrent exhibition of Dex700 and ICG signals remained stable until chronic to long‐standing stages. ICG imaging corresponded to the clinical arthritis scores; therefore, synovitis imaging is expected to assess the stages and severity of arthritis in actual clinical settings. Together, the simultaneous dual‐channel imaging enabled the quantitative observation of the inflammatory response, specifically in the peripheral joint area.

The cartilage extracellular matrix is a complex structure where several molecules interact to form structural and functional units, and this is of importance for RA imaging. The clinical use of the minimum joint space width and cartilage volume has been limited to the longitudinal measurement of disease progression (i.e., structural change over time). Moreover, although the shape of the articular cartilage can be differentiated by ultrasonography or MRI, no method that allows direct imaging of cartilage tissue by using specific contrast agents is available.^26^, ^27^ C700‐OMe reaches the cartilage matrix through diffusion‐based penetration, and binds to GAG and collagen through strong charge interactions.^10^ The C700‐OMe uptake was high in the cartilage of the wrist, ankle, and digits, but it was low in the destructed cartilage due to the lack of matrix components.^4^ Although the quantification is affected by pannus invasion in the acute stage, the clinical implication of using C700‐OMe is worthwhile, because direct anatomical imaging of cartilage damage could be achieved in the narrow joint space in the chronic stage of RA.

In this study, the most dramatic change was observed by imaging the bones using P800SO3. P800SO3 is primarily uptaken by HA because of phosphonate groups, which also allow strong binding to CP.^12^ In other words, P800SO3 can highlight neo‐ossification, where HA is formed, and bone resorption where CP is released. In fact, endosteal bone resorption by joint inflammation invokes the compensatory action of cortical remodeling mechanism to form periosteal bone in RA.^21^ This actually triggered amplified binding of P800SO3 to the periosteal bones (D28‐D25). Moreover, activated osteoclasts were also involved in the direct uptake of P800SO3, leading to a stronger fluorescence intensity in the immune prevailed region.^28^, ^29^ Nevertheless, this study has several challenges and limitations with respect to detectability and quantification. The intraoperative NIR imaging system is fundamentally limited to ≈<1 cm imaging depth because of tissue absorption and scatter. However, the tissues affected by RA are usually superficial joints, including the knee, wrist, and finger joints, which are easily accessed by the open‐air NIR imaging system without surgical exposure. For deep tissue imaging, our laboratory has also been developing several endoscopic/laparoscopic imaging systems capable of assessing almost any body space.^30^, ^31^ In addition, using the second NIR window (1000–1700 nm), we can reduce tissue scattering significantly, which improves image quality considerably.^32^, ^33^

In summary, the ability to visualize RA tissues by using tissue‐specific contrast agents suggests their diagnostic use in monitoring structural distortion and disease progression in real time. In addition, the quantitative analysis of progressive data sets can be used to lay the foundation of new treatment plans in pursuit of clinical remission. To understand the mechanism of action, however, the manipulated cells and tissues by each contrast agent can be further studied with supporting immunoassays, such as cytokine release test from primary cultured fibroblasts/immune cells and cell population measurement in the agent‐treated synovial tissues.

## 4 Experimental Section

*NIR Fluorophores*: ICG was purchased from Pulsion Medical Systems (Feldkirchen, Germany), and the injection sample was prepared using the manufacturer's diluent at a concentration of 3.2 × 10^−3^ m. C700‐OMe, P800SO3, PAM‐ZW800‐1, and ZW700‐1a *N*‐hydroxysuccinimide (NHS) ester were synthesized, as described in our previous reports,^10^, ^12^, ^34^, ^35^ and dissolved in dimethyl sulfoxide (DMSO) to make 10 × 10^−3^ m stock solutions. BoneTag (LI‐COR, Lincoln, NE, USA) was prepared in DMSO as a 10 × 10^−3^ m stock. Dex700 was prepared by conjugating ZW700‐1a on dextran using conventional NHS chemistry. Briefly, amino‐dextran70K (Invitrogen, Grand Island, NY, USA) was dissolved in phosphate‐buffered saline (pH 8 at a concentration of 1 × 10^−3^ m), and simply mixed with ten equivalent of ZW700‐1a NHS ester. After 1 h of rigorous shaking, the conjugate was purified by dialysis over 24 h followed by filtration using a mini‐P6 size exclusion column (Bio‐Rad, Hercules, CA, USA). The final extract was lyophilized to form a solid compound (93% yield; 0.98 conjugation ratio). The Dex700 injection solution was prepared in saline at a 1 × 10^−3^ m concentration.

*Optical and Physicochemical Property Analysis*: Optical properties of NIR fluorescent contrast agents were measured in 100% fetal bovine serum (FBS) supplemented with 50 × 10^−3^ m 4‐(2‐hydroxyethyl)‐1‐piperazineethanesulfonic acid (HEPES), pH 7.4. Absorbance and fluorescence emission spectra of the series of NIR fluorophores were measured using fiber optic HR2000 (200–1100 nm) spectrometers (Ocean Optics Inc., Dunedin, FL, USA). NIR excitation was provided by using 5 mW of a 655 nm red laser pointer (Opcom Inc., Xiamen, China) and 8 mW of a 765 nm NIR laser diode light source (Electro Optical Components Inc., Santa Rosa, CA, USA) coupled through a 300 µm core diameter, NA 0.22 fiber (Fiberguide Industries Inc., Stirling, NJ, USA). For fluorescence QY measurements, oxazine 725 in ethylene glycol (QY = 19%) and ICG in DMSO (QY = 13%) were used as calibration standards, under conditions of matched absorbance at 655 and 765 nm, respectively.^28^ Physicochemical properties, such as the molecular weight (Da), partition coefficient (log*D* at pH 7.4), and total polar surface area were calculated using Marvin and JChem calculator plugins (ChemAxon, Budapest, Hungary).

*In Vitro Cell Assay*: B16F10 murine melanoma cells, RAW264.7 murine macrophages, and MC3T3‐E1 mouse osteoblastic cells were purchased from ATCC (Manassas, VA, USA). Osteoclasts were differentiated from RAW264.7 cells by stimulation with 35 ng mL^−1^ RANKL (R&D Systems, Minneapolis, MN, USA). Simulation was repeated three times over 5 d. Cells were maintained in either Dulbecco's modified Eagle medium (DMEM) (for B16F10 and RAW264.7) or α‐MEM (for MC3T3‐E1 and differentiated osteoclasts), supplemented with 10% FBS (Gibco, Grand Island, NY, USA), 100 U mL^−1^ of penicillin, and 100 µg mL^−1^ of streptomycin (Gibco) at 37 °C in a humidified 5% CO_2_ atmosphere. When the cells reached 70%–80% confluence, P800SO3 was added to the dishes at 2 × 10^−6^ m in media. After 1 h incubation at 37 °C, cells were washed twice with media, and the NIR fluorescence images were obtained using a multi‐channel fluorescence microscope (see below).

*Intraoperative Optical Imaging System*: The basic design and setting of our dual‐NIR channel imaging system have been described in detail previously.^36^ In this study, 1 mW cm^−2^ of a 660 nm excitation light and 3.6 mW cm^−2^ of a 760 nm excitation light were used with white light (400–650 nm) at 5500 lux. Simultaneous color images (512 × 512 pixels) with the choice of either 700 or 800 nm fluorescence images were acquired using an AD‐130GE camera (JAI, Yokohama, Japan) installed with custom dual bandpass prism (channel 1: 710/50; channel 2: 780lp).^31^ The imaging system was remotely controlled by custom software at rates up to 15 Hz, except for field of view that was manually adjusted by a 3CCD zoom lens (Goyo Optical Inc., Saitama, Japan). In the color‐NIR merged image, 700 nm fluorescence and 800 nm fluorescence were pseudocolored red and green, respectively. The imaging head was positioned at a distance of 9 in. from the surgical field, and all NIR fluorescence images had identical exposure times and normalizations.

*Micro‐CT Measurement*: Micro‐CT images were acquired using a NanoPET/CT scanner that was equipped with a Gd202S X‐ray detector (Bioscan, Washington, DC, USA). CT scanning was performed at 45 kVp, 180 µA, and 240 projection/rotation using a complementary metal‐oxide‐semiconductor (CMSO) detector with a 37 µm pixel size. Projected data were reconstructed by setting the voxel size to 100 µm in VivoQuant software (InviCRO, Boston, MA, USA).

*Animal Models*: Animals were housed in an AAALAC‐certified facility and were studied under the supervision of BIDMC IACUC in accordance with approved institutional protocols (#057‐2014). Male DBA/1J mice weighing ≈25 g purchased from Jackson Laboratory (Bar Harbor, ME, USA) were anesthetized with 100 mg kg^−1^ of ketamine and 10 mg kg^−1^ of xylazine intraperitoneally (Webster Veterinary, Fort Devens, MA, USA). To generate CAIA models, DBA/1J mice were induced by a single intravenous injection of 2 mg of Arthrogen‐CIA arthritogenic monoclonal antibody cocktail (Chondrex, Redmond, WA, USA) on D0, followed by intraperitoneal injections of 50 µg lipopolysaccharide on D3, D14, and D21.^15^ Clinical development of arthritis in the paws was assessed by the arthritis score: 0, normal; 1, erythema; 2, erythema and mild swelling extending from the ankle to the tarsals; 3, erythema and moderate swelling extending from the ankle to the metatarsal joints; and 4, erythema and severe swelling encompassing the ankle, foot, and digits, or ankylosis of the limb. The total score per mouse ranged between 0 and 16.^15^

*Histology and NIR Fluorescence Microscopy*: Bone and cartilage tissues from normal and CAIA mice were preserved for histological microscopic assessment. Tissues extracted from the mice post‐intraoperative imaging were placed in 4% formalin solution for 1 d, followed by 5 d incubation in 0.5 m EDTA solution for decalcification. Frozen samples were made by mounting in Tissue‐Tek OCT compound (Fisher Scientific, Pittsburgh, PA, USA) and flash frozen in liquid nitrogen. Joint tissue samples from normal and CAIA mice were also embedded in paraffin. After sectioning (15 µm) each sample, the slices were observed by fluorescence microscopy, and then they were separately stained with hematoxylin‐eosin, Alcian blue–Safranin O, or the Von Kossa kit (Abcam, Cambridge, MA, USA). NIR fluorescence microscopy was performed on a 6‐channel Nikon Eclipse TE2000 microscope system equipped with a 75 W Xenon light source, Orca‐ER camera (Hamamatsu, Bridgewater, NJ, USA), and NIR‐compatible 10 × and 40 × Plan Fluor objective lens (Nikon, Melville, NY, USA). Two custom filter sets (Chroma, Brattleboro, VT, USA) composed of 650 ± 22 and 750 ± 25 nm excitation filters, 675 and 785 nm dichroic mirrors, and 710 ± 25 and 810 ± 20 nm emission filters were, respectively, used to detect C700‐OMe (pseudocolored in red) and P800SO3 (pseudocolored in green) emissions.

*Post‐Image Analysis*: All post‐image analysis and quantification were performed using ImageJ 1.48 (NIH, Bethesda, MD, USA) and Microsoft Excel (Redmond, WA, USA). The fluorescence and background intensity of an ROI over each tissue were quantified using SBR, which was calculated as SBR = fluorescence/background (background = signal intensity of the blank area outside of the tissue). All NIR fluorescence images were obtained using the same exposure times and normalization for each fluorophore. To compare cartilage destruction, an intensity line profile of C700‐OMe preceded the image of the wrist and ankle area at each time point. Bone signal distribution was quantified by sequential procedures of segmentation, which was completed through ROI selection, static thresholding based on SBR, binary image, and calculation of area fraction. Results were representative of at least three independent experiments and presented as a mean ± standard deviation (s.d.). Curve fitting was performed using Prism, version 6.0 software (GraphPad, San Diego, CA, USA), and one‐way ANOVA followed by the Tukey multiple comparison test was used to assess statistical differences among multiple groups. A *P* value < 0.05 was considered significant: *P < 0.05, **P < 0.01, and ***P < 0.001.

## Conflict of Interest

The authors declare no conflict of interest.

## Author Contributions

J.H.L. and S.Y.J. contributed equally to this work. S.Y.J., J.H.L., G.K.P., K.B., and H.H. performed the experiments. S.Y.J., J.H.L., G.E.F., and H.S.C. reviewed, analyzed, and interpreted the data. S.Y.J., J.H.L., and H.S.C. wrote the paper. All authors discussed the results and commented on the manuscript.

## Supporting information

Supporting Information
