# Supplementary material for: Fluorometric Imaging for Early Diagnosis and Prognosis of Rheumatoid Arthritis
Source: Adv Sci (Weinh). 2019 Dec 1;7(1):1902267. doi: 10.1002/advs.201902267 (PMC6947695; doi:10.1002/advs.201902267)
Supplement: Supplementary file 1 — Supporting Information [file ADVS-7-1902267-s001.pdf]

Copyright WILEY-VCH Verlag GmbH & Co. KGaA, 69469 Weinheim, Germany, 2019.

Supporting Information

for *Adv. Sci.,* DOI: 10.1002/advs.201902267

Fluorometric Imaging for Early Diagnosis and Prognosis of Rheumatoid Arthritis

Jeong Heon Lee, Sang Youn Jung, G. Kate Park, Kai Bao, Hoon Hyun, Georges El Fakhri, and Hak Soo Choi**

Supporting Information

Fluorometric Imaging for Early Diagnosis and Prognosis of Rheumatoid Arthritis

Jeong Heon Lee[1,*], Sang Youn Jung[2,*,**], G. Kate Park[1], Kai Bao[1], Hoon Hyun[3], Georges El Fakhri[1], and Hak Soo Choi[1,**]

[1]Gordon Center for Medical Imaging, Department of Radiology, Massachusetts General Hospital and Harvard Medical School, Boston, MA 02114, USA

[2]Division of Rheumatology, Department of Internal Medicine, CHA Bundang Medical Center, CHA University, Seongnam 13496, South Korea

[3]Department of Biomedical Sciences, Chonnam National University Medical School, Gwangju 501-746, South Korea

[*]These authors contributed equally to this work.

Correspondence to: S.Y.J. (jungsy7597@cha.ac.kr) or H.S.C. (hchoi12@mgh.harvard.edu)

The PDF file includes:

Table S1. Physicochemical and optical properties of targeted NIR fluorophores.

Figure S1. 2D/3D structure and absorbance and fluorescence spectra of NIR fluorophores.

Figure S2. Biodistribution of Dex700, C700-OMe, and P800SO3 in CD-1 mice.

Figure S3. Synovitis imaging of mouse hindpaw and SBR chart.

Figure S4. Soft tissue swelling in the forepaw and hindpaw of mice with CAIA.

Figure S5. Histologic images of resected knee and ankle tissues from normal mice and mice with CAIA.

Figure S6. Differential specificity of bone-specific NIR fluorophores.

Figure S7. Micro-CT and NIR fluorescence imaging of mouse forepaw.

Figure S8. Binary images of P800SO3 signal distribution of the mouse forepaw in RA progression.

Figure S9. Real-time intraoperative NIR fluorescence imaging of mouse knee joint.

Table S1. Physicochemical and optical properties of targeted NIR fluorophores.

Property	ZW700-1a	ICG	C700-OMe	P800SO3
Molecular Weight (Da)	877.12	751.97	615.91	986.98
LogD at pH 7.4	-4.34	4.91	-4.93	-7.03
TPSA (Å^2)	170.01	120.65	24.71	285.07
ε ($M^{-1}cm^{-1}$) in FBS	205,500	121,000	96,500	210,000
λ_{exc} (nm)	653	807	666	785
λ_{em} (nm)	672	822	692	802
Stokes Shift (nm)	19	15	26	17
Φ (%)	19.4	9.3	9.7	15.1

In silico molecular properties were calculated using Marvin and JChem calculator plugins. All optical properties were measured in 100% FBS buffered with 50 mM HEPES, pH 7.4.

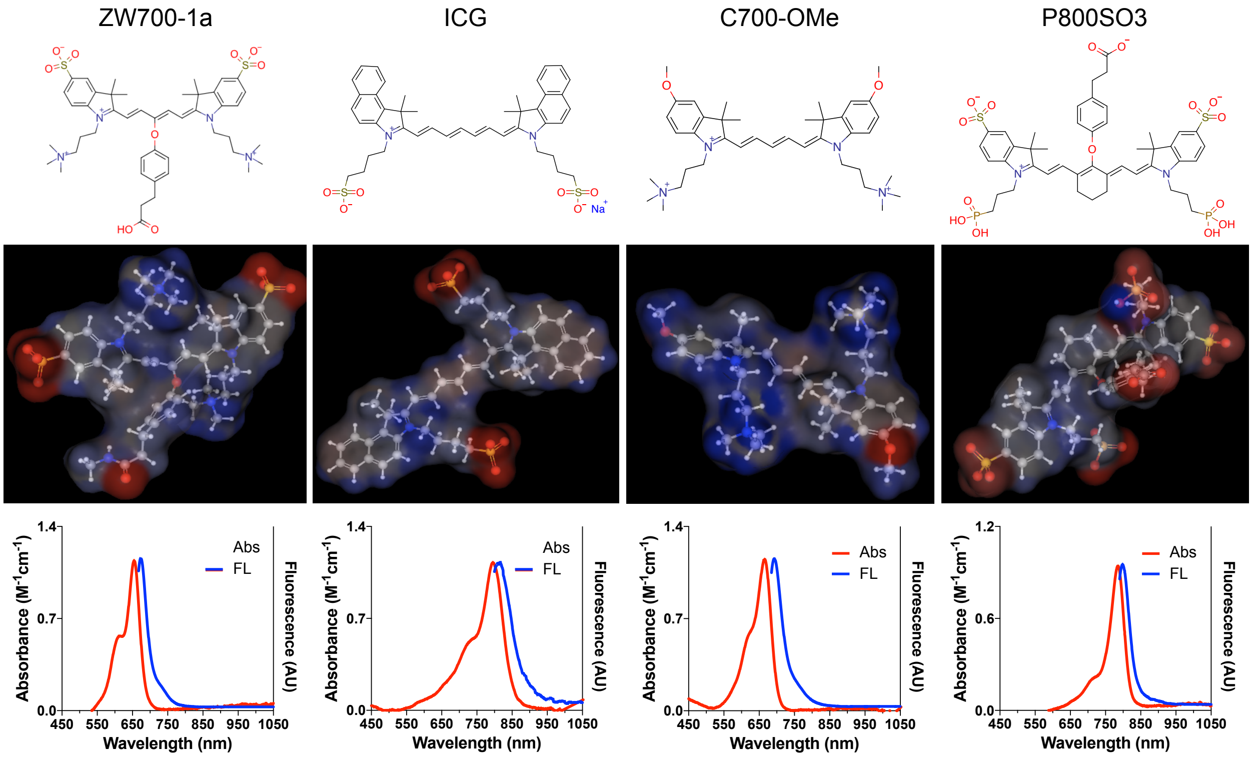

Figure S1. 2D/3D structure of each NIR fluorophore (top) and their absorbance and fluorescence emission spectra in 100% FBS supplemented with 50 mM HEPES, pH 7.4 (bottom).

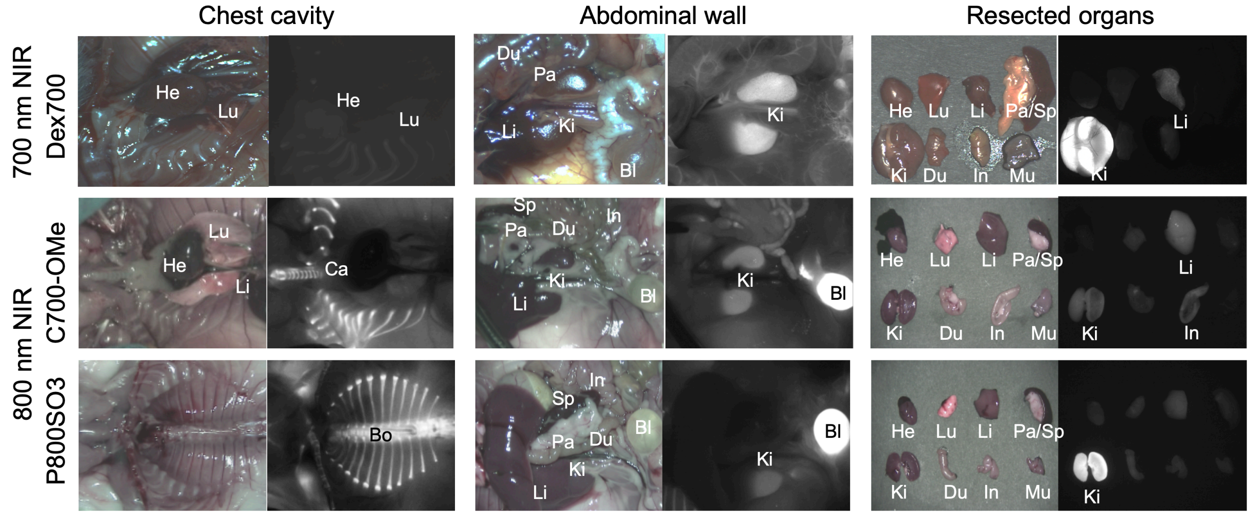

Figure S2. Biodistribution of Dex700, C700-OMe, and P800SO3. 25 nmol of each agent was injected intravenously into CD-1 mice 4 h prior to imaging. Abbreviations used are: Bl, bladder; Bo, bone; Ca, cartilage; Du; duodenum; He, heart; In, intestine; Ki, kidney; Li, liver; Lu, lung; Mu, muscle; Pa, pancreas; Sp, spleen.

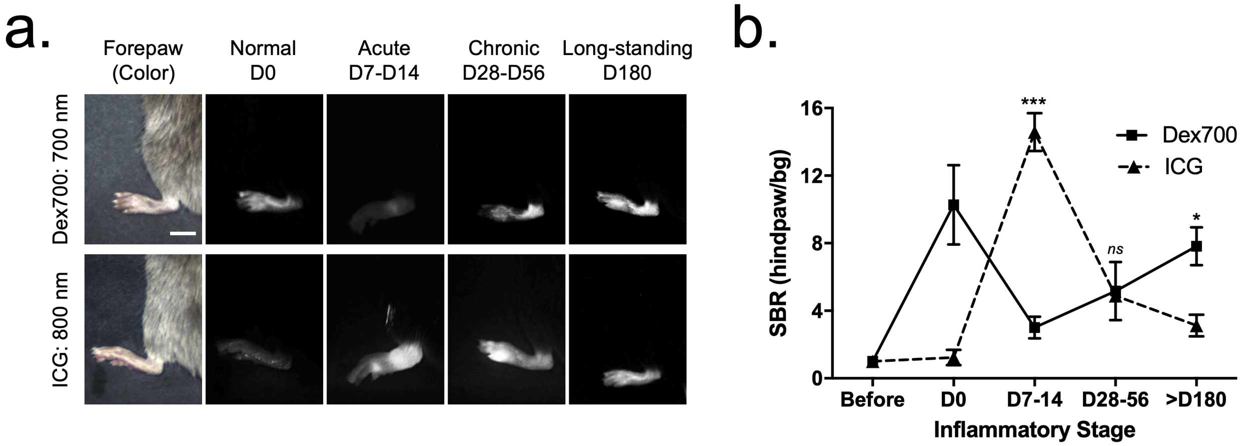

Figure S3. a) *In vivo* imaging of mouse hindpaws after the intravenous injection of Dex700 (25 nmol, red color) and ICG (50 nmol, green color) 30 min prior to imaging. Scale bars = 1 cm. Images are representative of independent experiments (n = 3). **b**) Quantitative scoring (SBR) charts of Dex700 and ICG for D180 in the inflammatory stages. SBR is calculated by the fluorescence intensity of the hindpaw versus the background (bg) signal of the neighboring blank area (n = 3, mean ± standard deviation; *$P < 0.05$, ***$P < 0.001$, ns: not significant).

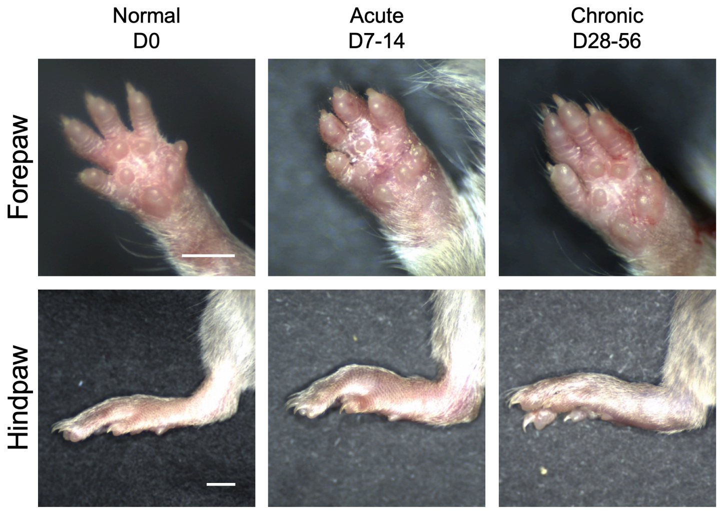

Figure S4. Soft tissue swelling in the forepaw (top) and hindpaw (bottom) of mice with CAIA over 2 months of RA progression. Scale bars = 3 mm.

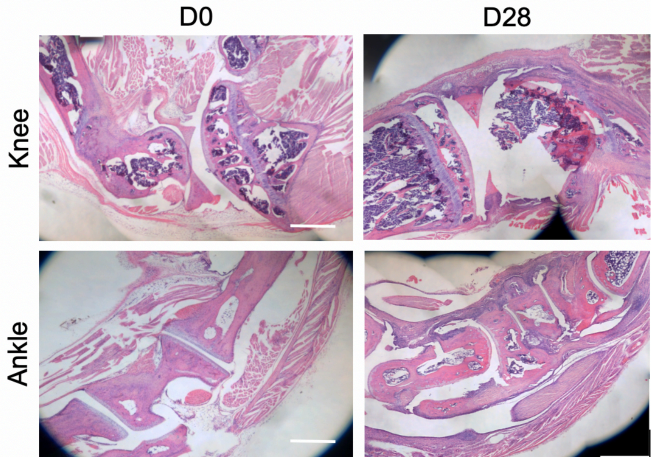

Figure S5. Histologic H&E images of resected knee (top) and ankle (bottom) tissues from normal mice (D0) and mice with CAIA (D28). Scale bars = 200 μm.

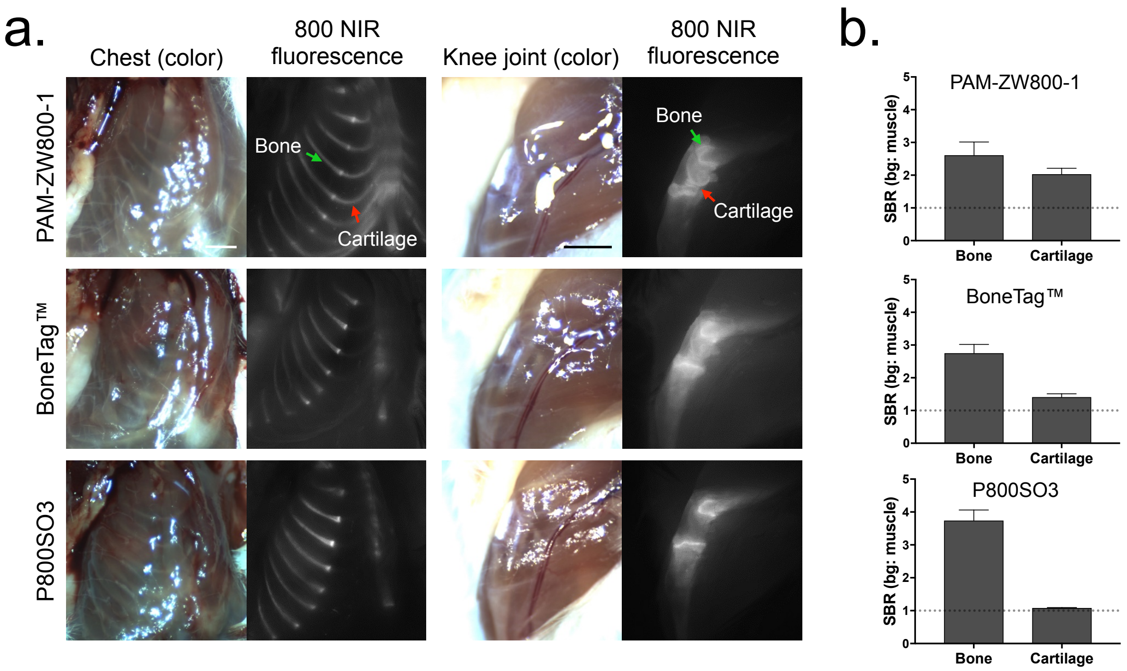

Figure S6. a) Tissue targetability of bone-specific NIR fluorophores. 25 nmol (0.05 mg/kg) of PAM-ZW800-1, BoneTag, or P800SO3 was injected into CD-1 mice 24 h prior to imaging. Cartilage and bone of central skeleton in the chest area (left) and knee joint area (right) were observed. Scale bars = 3 mm. **b**) SBR chart of bone and cartilage signals from each fluorophore.

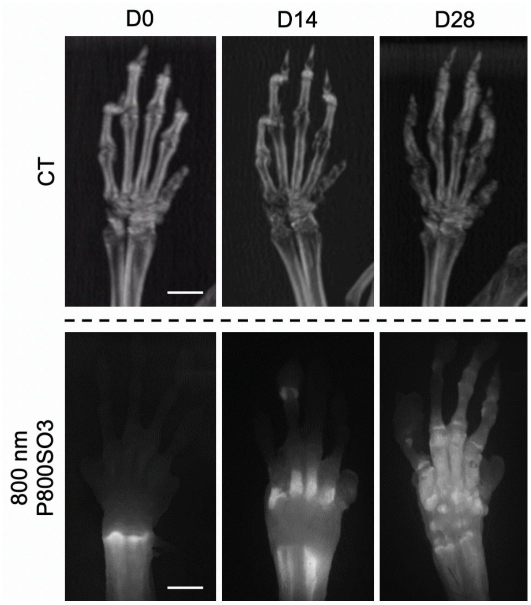

Figure S7. Micro-CT (top) and NIR fluorescence (bottom) images of mouse forepaw over 1 month of RA progression. 25 nmol (0.05 mg/kg) of P800SO3 was injected intravenously into mice with CAIA 24 h prior to imaging. Scale bars = 3 mm.

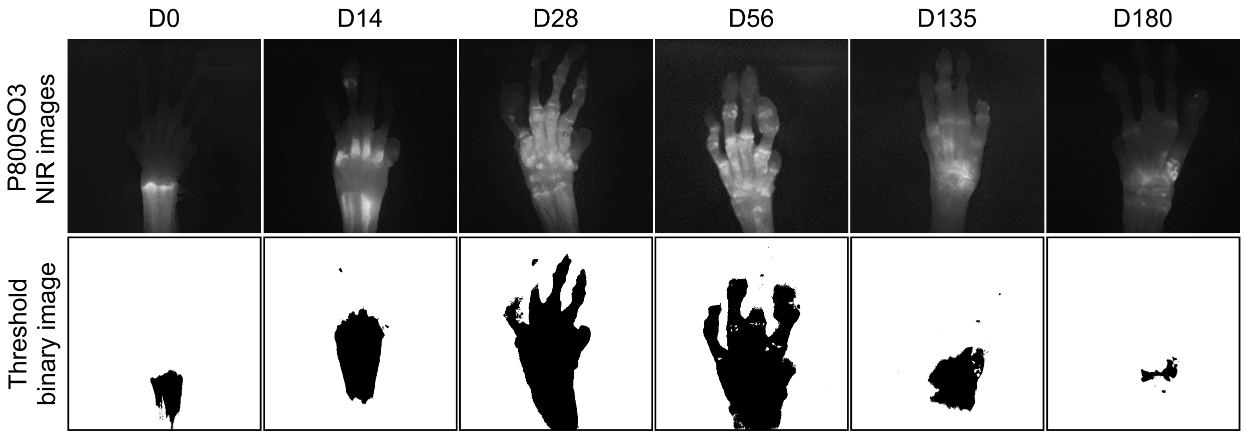

Figure S8. Signal distribution of P800SO3 in mouse forepaw over 6 months of RA progression (top), and each binary image in the same ROI after background thresholding for the image segmentation (bottom).

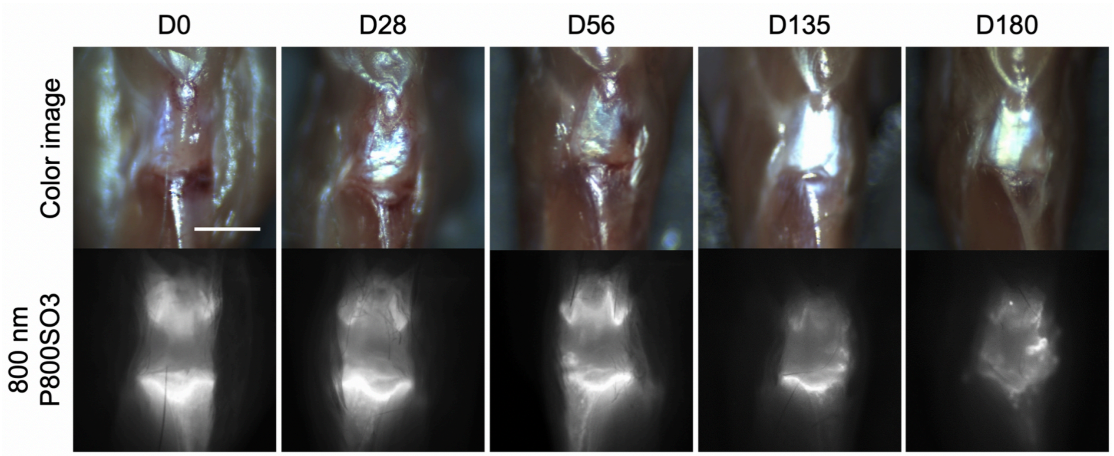

Figure S9. Real-time intraoperative NIR fluorescence images of mouse knee joint over 6 months of RA progression. 25 nmol (0.05 mg/kg) of P800SO3 was injected intravenously into mice with CAIA 24 h prior to imaging. Scale bar = 3 mm.
